# Lactoferrin-Hexon Interactions Mediate CAR-Independent Adenovirus Infection of Human Respiratory Cells

**DOI:** 10.1128/JVI.00542-20

**Published:** 2020-07-01

**Authors:** B. David Persson, Annasara Lenman, Lars Frängsmyr, Markus Schmid, Clas Ahlm, Andreas Plückthun, Håvard Jenssen, Niklas Arnberg

**Affiliations:** a Division of Virology, Department of Clinical Microbiology, Umeå University, Umeå, Sweden; b Institute for Experimental Virology, TWINCORE, Centre for Experimental and Clinical Infection Research, Hannover, Germany; c Department of Biochemistry, University of Zurich, Zurich, Switzerland; d Department of Science and Environment, Roskilde University, Roskilde, Denmark; University of Texas Southwestern Medical Center

**Keywords:** adenovirus, CAR, cellular receptor, lactoferrin, tropism

## Abstract

Virus entry into host cells is a complex process that is largely regulated by access to specific cellular receptors. Human adenoviruses (HAdVs) and many other viruses use cell adhesion molecules such as the coxsackievirus and adenovirus receptor (CAR) for attachment to and entry into target cells. These molecules are rarely expressed on the apical side of polarized epithelial cells, which raises the question of how adenoviruses—and other viruses that engage cell adhesion molecules—enter polarized cells from the apical side to initiate infection. We have previously shown that species C HAdVs utilize lactoferrin—a common innate immune component secreted to respiratory mucosa—for infection via unknown mechanisms. Using a series of biochemical, cellular, and molecular biology approaches, we mapped this effect to the proteolytically cleavable, positively charged, N-terminal 49 residues of human lactoferrin (hLF) known as human lactoferricin (hLfcin). Lactoferricin (Lfcin) binds to the hexon protein on the viral capsid and anchors the virus to an unknown receptor structure of target cells, resulting in infection. These findings suggest that HAdVs use distinct cell entry mechanisms at different stages of infection. To initiate infection, entry is likely to occur at the apical side of polarized epithelial cells, largely by means of hLF and hLfcin bridging HAdV capsids via hexons to as-yet-unknown receptors; when infection is established, progeny virions released from the basolateral side enter neighboring cells by means of hLF/hLfcin and CAR in parallel.

**IMPORTANCE** Many viruses enter target cells using cell adhesion molecules as receptors. Paradoxically, these molecules are abundant on the lateral and basolateral side of intact, polarized, epithelial target cells, but absent on the apical side that must be penetrated by incoming viruses to initiate infection. Our study provides a model whereby viruses use different mechanisms to infect polarized epithelial cells depending on which side of the cell—apical or lateral/basolateral—is attacked. This study may also be useful to understand the biology of other viruses that use cell adhesion molecules as receptors.

## Abstract (precis)

Many viruses enter target cells using cell adhesion molecules as receptors. Paradoxically, these molecules are abundant on the lateral and basolateral side of intact, polarized, epithelial target cells, but absent on the apical side that must be penetrated by incoming viruses to initiate infection. Our study provides a model whereby viruses use different mechanisms to infect polarized epithelial cells depending on which side of the cell—apical or lateral/basolateral—is attacked. This study may also be useful to understand the biology of other viruses that use cell adhesion molecules as receptors.

## INTRODUCTION

Epithelial tissues consist of multiple layers of epithelial cells, which are tightly connected to each other by homo- or heterodimeric cell adhesion molecules. Several unrelated viruses infecting epithelial tissues have evolved to use these cell adhesion molecules as receptors for attachment to and/or entry into epithelial cells (1, 2). This is puzzling, given that these receptor molecules usually localize below tight junctions (3) and are not accessible from the apical side of intact polarized epithelial cells. It is therefore unclear how viruses that use cell adhesion molecules as receptors penetrate intact epithelial cell layers from the apical surface, e.g., when incoming viruses initiate infection of a healthy tissue, but also when progeny virions escape from the infected cell to the apical surface and infect cells distant from the original infected cell.

Human adenoviruses (HAdVs) are common human pathogens that normally cause self-limiting infections in healthy adults. In children under the age of five, respiratory HAdVs are responsible for up to 15% of all lower respiratory tract infections (4–6). Species C HAdVs, including HAdV-C5, also cause persistent infections in tonsils and adenoids (7). Progeny virions can be shed for several months (8, 9), and such persistent infections can develop into severe, systemic, and even fatal infections in immunocompromised patients (10).

Most HAdVs (including HAdV-C5) use the cell adhesion molecule coxsackievirus and adenovirus receptor (CAR) as a receptor for infection of target cells (2, 11, 12). Intriguingly, like most other cell adhesion molecules, CAR is not, or is rarely, expressed on the apical surface of polarized epithelial cells (13–18). CAR can be used both as an entry receptor and for enhanced escape of progeny virions from an infected tissue, mediated by production and basolateral secretion of excess CAR-binding fibers capable of resolving intercellular CAR homodimers (19). However, this does not explain how viruses approaching a noninfected tissue from the apical side initiate infection. To some extent, this can be explained by upregulation of CAR by the proinflammatory cytokine interleukin 8 (IL-8) (20, 21), but this mechanism does not explain how infection is initiated in the absence of preexisting inflammation.

We have previously demonstrated that lactoferrin (hLF) acts as a proviral component that efficiently increases species C HAdV infection (15). This is surprising, as hLF usually exerts antimicrobial functions (22–24), as well as preventing virus attachment to target cells and/or inhibiting virus replication (25–27). hLF is produced largely by neutrophils, but also by primary epithelial cells, and it is naturally present in a vast majority of body fluids in concentrations of up to several mg/ml, where it protects epithelia from pathogens (28–31). Once present in the mucosa, secreted hLF is cleaved by cellular proteases to release the highly positively charged, N-terminal, 49-residue peptide of hLF named human lactoferricin (hLfcin), which comprises the main antimicrobial and immune system-stimulating properties of hLF (32–35).

## RESULTS

### Human lactoferricin is required for efficient HAdV-C5 transduction of human respiratory epithelial cells.

**FIG 1:**
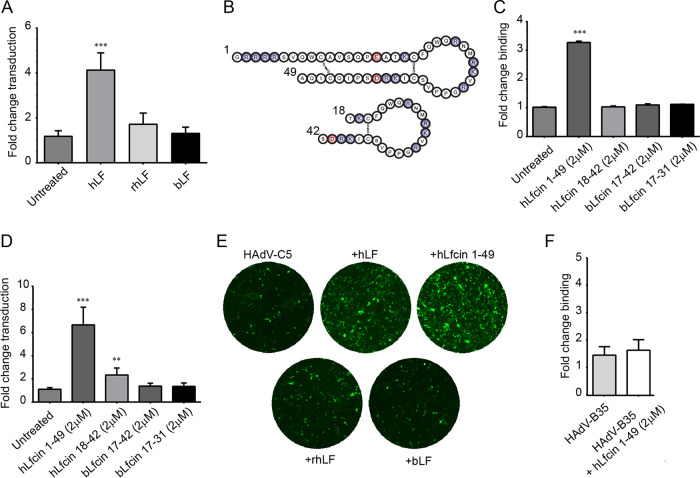
Effects of LF and Lfcin on HAdV transduction of and binding to A549 cells. (A) HAdV-C5 transduction of A549 cells after preincubation with LF of different sources (hLF, purified human LF; rhLF, recombinant human LF; bLF, purified bovine LF). (B) Schematic drawing of human Lfcin peptides 1-49 and 18-42. Positively charged amino acids are shown in blue, and negatively charged amino acids in shown in red. (C) Binding to and (D) transduction of A549 cells by HAdV-C5 after preincubation with human and bovine Lfcin-derived peptides. (E) Example images of transduced A549 cells used for quantification. (F) HAdV-B35 virion binding to A549 cells after preincubation with hLfcin1-49. Data are presented as means ± standard error of the mean (SEM) from 3 to 5 independent experiments. Statistical significance was determined with two-way analysis of variance (ANOVA) (A, C, D), or Student’s *t* test (F); **, *P* < 0.01; ***, *P* < 0.001.

**FIG 2:**
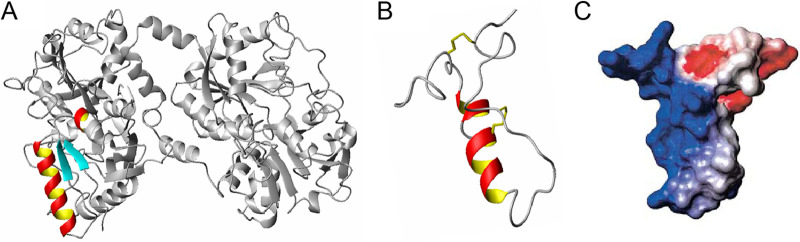
The structures of hLF and hLfcin1-49. (A) Human lactoferrin (PDB code 1LFG; deposited by Haridas et al. [63]) with the hLfcin1-49 peptide indicated in yellow/red/cyan as in Gifford et al. (64). (B) hLfcin1-49 after pepsin cleavage (PDB code 1Z6V; deposited by Hunter et al. [65]). (C) Charge distribution of hLfcin1-49 from Gifford et al. (64). Negatively charged amino acids are colored red, and positively charged amino acids are blue.

We have previously shown that all species C HAdVs infect epithelial and T cells more efficiently in the presence of hLF (15). In this study, we investigated the molecular mechanisms behind hLF-dependent infection. As a first step, we investigated the structural features whereby hLF mediates HAdV-C5 infection of human respiratory A549 cells. In total, we used three different types of lactoferrin, namely, hLF purified from human tear fluid (present in several mg/ml), a recombinant form of hLF produced in Aspergillus with a human amino acid backbone but with a high-mannose glycosylation pattern (rhLF), and bovine lactoferrin (bLF) from cow milk. Preincubation of HAdV-C5_GFP vectors with 1 mg/ml hLF increased HAdV-C5_GFP transduction of human respiratory epithelial A549 cells 4-fold (Fig. 1A and E). Neither bLF nor rhLF affected HAdV-C5_GFP transduction at the same concentration, indicating that both glycans and the amino acid sequence may contribute to the effect. Next, we wanted to investigate if the antimicrobial peptide of hLF, hLfcin, could mediate HAdV-C5 infection on its own. hLfcin1-49, with the natural hairpin form of hLfcin (Fig. 1B and 2), was used at 2 μM concentration (corresponding to about 1 mg/ml of hLF) and promoted both viral binding to and transduction of A549 cells (by 3- and 6-fold, respectively; Fig. 1C to E), using the lowest concentration that resulted in a maximal effect. bLF and other (bovine or human) Lfcins (bLfcin17-31, bLfcin17-42, and hLfcin18-42) had no or little effect (Fig. 1C and D) at this concentration. Furthermore, hLfcin1-49 had no, or minimal, effect on HAdV-B35 binding (Fig. 1F). These results suggest that hLfcin1-49 also mediates HAdV-C5 binding and transduction and is responsible for hLF-dependent HAdV-C5 binding to and transduction of A549 cells.

### hLfcin-mediated HAdV-C5 transduction is independent of CAR and integrins.

**FIG 3:**
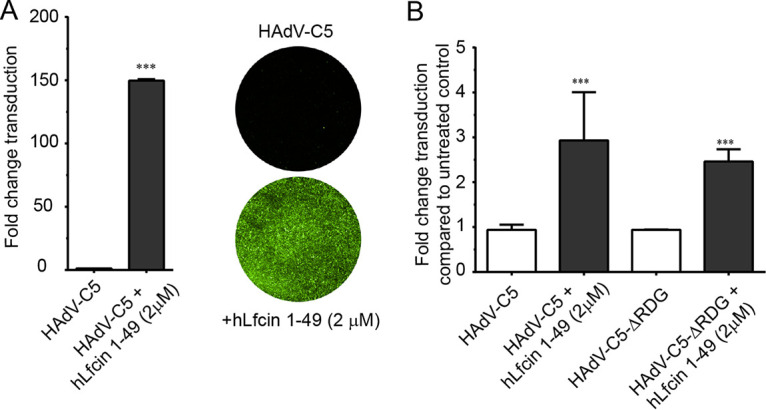
Impact of the CAR and integrins on hLfcin1-49-mediated transduction. (A) HAdV-C5 transduction of human integrin-negative and human CAR-negative CHO-K1 cells after preincubation with hLfcin1-49, including example images of HAdV-C5 transduction of CHO-K1 cells after preincubation with/without hLfcin1-49. (B) HAdV-C5 transduction of A549 cells by HAdV-C5 and HAdV-C5 ΔRGD, after preincubation with hLfcin1-49. Data are presented as means ± SEM from 3 independent experiments. Statistical significance was determined with two-way ANOVA (B) or Student’s *t* test (A); ***, *P* < 0.001.

Adenoviruses are icosahedral viruses composed of three major capsid proteins, namely, hexon, penton base, and fiber. The current model for adenovirus entry into epithelial cells is largely based on nonpolarized cell lines such as A549, in which CAR is abundant and exposed on the entire plasma membrane (36) and where primary attachment is mediated by the CAR-binding protruding knob of the fiber protein. This is followed by interactions between the RGD motif in the penton base and integrins, resulting in endocytosis (37, 38). To investigate if hLfcin1-49-mediated entry of HAdV-C5_GFP depends on CAR and/or integrins, we quantified transduction of Chinese hamster ovary cells (CHO) cells, as these cells lack human CAR and integrins and are almost completely refractory to HAdV transduction/infection (39). Interestingly, preincubation with hLfcin1-49 increased transduction at least 150-fold (Fig. 3A). In fact, the transduction was enhanced so much that a quantification was challenging due to a close to 100% transduction of cells with oversaturated pixels in the presence of hLfcin1-49. We could not lower the viral dose, since this resulted in no infection of untreated control cells. This suggested that human CAR and integrins are not essential for HAdV-C5 transduction in the presence of hLfcin1-49. hLfcin1-49 also enhanced transduction of green fluorescent protein (GFP)-encoding HAdV-C5 vectors with the integrin-binding RGD motif -HAIRGDTFAT- replaced with -TSRAEEK- (40) in A549 cells to a similar extent as that of the control HAdV-C5 vector (Fig. 3B). This further supports that hLfcin1-49-mediated transduction is independent of RGD-binding integrins. Basal, Lfcin-independent transduction of A549 cells was very high, which is probably an effect of the large amounts of CAR- and RGD-binding integrins on these cells. Still, hLfcin1-49 increased transduction 3-fold, suggesting that hLfcin1-49 plays an important role during HAdV-C5 infection of human respiratory epithelial cells, even in the presence of CAR and integrins.

### Structural determinants of hLfcin-mediated HAdV-C5 transduction.

**FIG 4:**
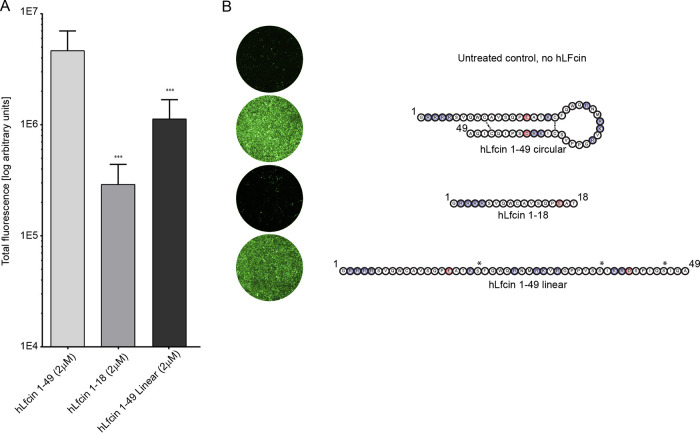
Effect of hLfcin length and structure on hLfcin-mediated HAdV-C5_GFP transduction of CHO-K1 cells. (A) Quantification of transduction. Data are presented as means ± SEM from 5 independent experiments. (B) Example images of transduced cells with the different variants of hLfcin. Cysteines mutated to serines are indicated by asterisks. Statistical significance comparing hLfcin1-49 with the altered types of hLfcin was determined with two-way ANOVA; ***, *P* < 0.001.

**FIG 5:**
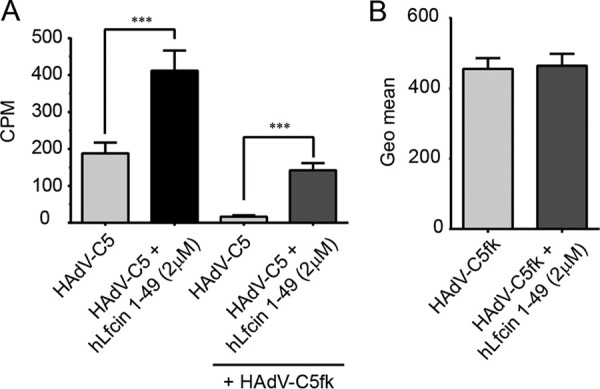
The fiber knob is not engaged in hLfcin1-49-mediated ^35^S-labeled HAdV-C5 binding to A549 cells. (A) hLfcin1-49-mediated HAdV-C5 virion binding to A549 cells preincubated with or without soluble HAdV-C5 fiber knob. (B) HAdV-C5 fiber knob (HAdV-C5fk) binding to A549 cells after preincubation with hLfcin1-49. Data are presented as means ± SEM from 3 independent experiments. Statistical significance was determined with two-way ANOVA (A) or Student’s *t* test (B); ***, *P* < 0.001.

We further hypothesized that the hairpin form of hLfcin1-49 bridges HAdV-C5 interactions with cellular receptors by anchoring the virus and the receptor with the opposite charges of hLfcin1-49 (Fig. 2C). To investigate this, and to pinpoint the structure and regions of hLfcin needed to bridge HAdV-C5 to cellular receptors, we compared the effect of the hairpin form with those of a linear form of hLfcin1-49 and a truncated linear peptide (hLfcin1-18). To ensure that the peptides remained linear, three out of the four cysteines were mutated to serines (Fig. 4B). All the different forms of hLfcin enhanced HAdV-C5 transduction of CHO-K1 cells, but there was a clear dependence on peptide length and structure (Fig. 4). We concluded that the maximal effect of Lfcin requires an intact structure (Fig. 2) and a full-length peptide. Next, we determined which HAdV-C5 capsid protein interacts with hLF and hLfcin1-49. The addition of soluble fiber knobs (2-fold more than in hLfcin1-49) did not outcompete hLfcin1-49-mediated HAdV-C5 virion binding to A549 cells (Fig. 5A). In fact, when soluble fiber knobs were added, basal binding decreased as expected due to the blockage of CAR. hLfcin1-49-mediated binding, on the other hand, increased substantially in relation to basal binding upon the addition of soluble fiber knobs. Thus, in the presence of soluble fiber knobs, the relative increase in hLfcin1-49-mediated virion binding became even more prominent. In addition, hLfcin1-49 failed to increase fiber knob binding to A549 cells (Fig. 5B). These results demonstrate that the fiber knob is not involved in hLfcin1-49-mediated HAdV-C5 binding to cells.

**FIG 6:**
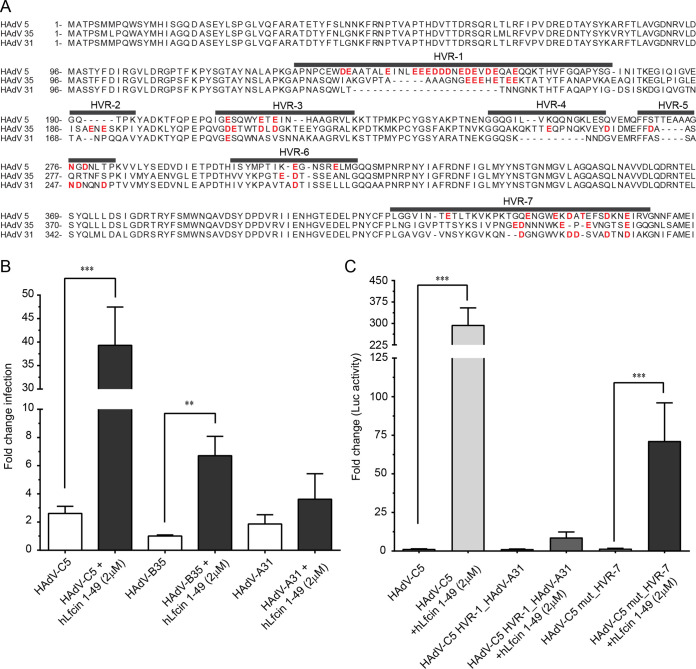
hLfcin1-49-mediated HAdV-C5 infection requires an intact HVR1 loop. (A) Sequence alignment of the hypervariable regions (dark gray lines) of HAdV-C5, HAdV-B35, and HAdV-A31 hexons. Negatively charged amino acids are colored red. (B) HAdV-C5, HAdV-B35 and HAdV-A31 infection of A549 cells in the presence of hLfcin1-49. (C) Effect of replacing HVR-1 in AdV-C5 with HVR-1 from AdV-A31, and effect of deleting HVR-7 in HAdV-C5 on transduction of CHO-K1 cells. Data are presented as means ± SEM from at least 3 independent experiments. Statistical significance was determined with two-way ANOVA; **, *P* < 0.01; ***, *P* < 0.001.

**FIG 7:**
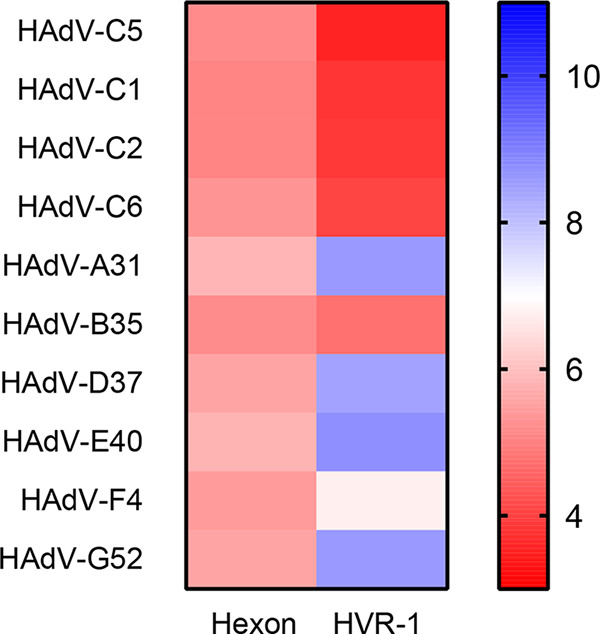
Analysis of hexon and HVR-1 pI value, comparing representative members of all adenovirus species (A, B, D, E, F, and G) to species C adenoviruses (HAdV-C1, HAdV-C2, HAdV-C5, HAdV-C6). Charge is displayed from a pI of 3 (red) to 11 (blue), with neutral pI at 7 (white).

The hexon hypervariable region-7 (HVR-7) of HAdV-C5 and HAdV-A31 interacts with human coagulation factor IX and X, which bridge virions to cellular heparan sulfate, resulting in enhanced infection of target cells (41, 42). Assuming that a similar mechanism is used by hLfcin, we aligned hexon amino acid sequences of HAdVs known to use hLF for enhanced infection (such as HAdV-C5), with those of HAdVs expected not to use hLF (such as HAdV-A31 and HAdV-B35). This revealed a unique stretch of acidic residues in HVR-1 of HAdV-C5 (Fig. 6A), which is also present in all other members of species C HAdVs, but not found, or is less pronounced, in other HAdVs (Fig. 7). These acidic residues reduce the pI locally around HVR-1 from greater than seven to around, or even below, four. We hypothesized that the reason for hLF to only enhance species C HAdV transduction (15) may be that the positively charged domain of hLfcin1-49 (Fig. 2) interacts with HVR-1 of these HAdVs better than with HAdVs of other clades, with a less pronounced negative charge (i.e., HAdV-B35) or with no obvious negative charge (i.e., HAdV-A31) in their respective HVR-1. To address the role of HVR-1, and to fully investigate the impact of hLfcin in HAdV multicycle infection of human respiratory cells, we infected A549 cells with these three wild-type HAdVs. Four days later, we collected supernatants, reinfected a fresh monolayer of A549 cells, and quantified infected cells. This setup likely mimics the *in vivo* situation better, even though we do not account for the loss in bioavailable hLfcin1-49 over time from uptake and degradation, as hLfcin1-49 is available also after infection and not only during infection. Interestingly, with hLfcin1-49 present, we noticed a significant increase in infection for both HAdV-C5 (40-fold) and HAdV-B35 (8-fold), but not for HAdV-A31 (Fig. 6B). Thus, even in A549 cells that have high levels of CAR, hLfcin increased HAdV-C5 infection substantially. We also conclude that even very subtle differences in infection observed after 44 h of transduction can translate into substantially larger effects over time, with potentially great biological and medical consequences. In addition, HAdVs beyond species C can also use hLfcin1-49 for enhanced infection of target cells, but to a lesser extent.

Encouraged by these results, we generated luciferase-encoding chimeric viruses in which HVR-1 of HAdV-C5 was replaced with the HVR-1 of HAdV-A31 (43). We also used viruses in which HVR-7 was altered (44), as HVR-7 of HAdV-C5 has previously been shown to engage coagulation factors (42, 45). To investigate the impact of this modification in a setting where CAR is not present, we turned back to CHO cells. As expected, hLfcin1-49 greatly (280-fold) enhanced transduction of CHO-K1 cells by HAdV-C5_Luc_GFP (Fig. 6C). However, when HVR-1 in HAdV-C5 was replaced with HVR-1 of HAdV-A31, the hLfcin1-49-mediated increased transduction was completely lost. We also noticed a 3-fold reduction in hLfcin1-49-mediated transduction of HAdV-C5_Luc_GFP with the HVR-7 loop altered. From these data, we conclude that hLfcin1-49-mediated HAdV infection of target cells requires a negatively charged hexon HVR-1, and we suggest that this occurs through a direct interaction between hexon HVR-1 and hLfcin1-49.

With a potential direct interaction between hexon protein and hLF or hLfcin1-49, we performed surface plasmon resonance analyses using purified hexons from HAdV-C5, HAdV-B35, and HAdV-A31 immobilized on the sensor chip surface. We observed three very different affinity levels between the HAdV types for both hLF and hLfcin1-49 (Table 1). The HAdV-C5 hexon bound most strongly to hLF (29 nM), about 5-fold more strongly than the HAdV-B35 hexon-hLF interaction (151 nM), and this interaction in turn was 5-fold stronger than the HAdV-A31 hexon-hLF interaction (827 nM). When hLF was replaced with hLfcin1-49, we observed a general decrease of affinities (lower μM range; Table 1), but again the binding was strongest to HAdV-C5, followed by that to HAdV-B35 and HAdV-A31. The spread in affinities was significantly larger, namely, an ∼30-fold difference between HAdV-C5 (0.73 μM) and HAdV-B35 (22.1 μM) and an 8.5-fold difference between HAdV-B35 and HAdV-A31 (187 μM). Collectively, these data suggest that the major anchoring point for hLF/hLfcin1-49 is located within HVR-1 of the hexon, where the relative acidity of HVR-1 determines the ability to use Lfcin for infection.

**TABLE 1:** Surface plasmon resonance analysis of HAdV hexon interactions with hLF and hLfcin1-49

Ligand	Analyte	Parameter^*a*^		
		*K_D_*	*k_a_* (M^−1^ · s^−1^)	*k_d_* (s^−1^)
HAdV-C5 hexon	hLF	29 ± 3.6 nM	5.3 × 10^4^ ± 2.6 × 10^3^	0.00154 ± 0.00024
HAdV-B35 hexon	hLF	151 ± 14 nM	2.5 × 10^4^ ± 2.5 × 10^3^	0.00354 ± 0.00014
HAdV-A31 hexon	hLF	827 ± 55 nM	1.2 × 10^4^ ± 1.1 × 10^3^	0.01011 ± 0.00024
HAdV-C5 hexon	hLfcin1-49	0.73 ± 0.206 μM	ND	ND
HAdV-B35 hexon	hLfcin1-49	22.1 ± 13.6 μM	ND	ND
HAdV-A31 hexon	hLfcin1-49	187 ± 155 μM	ND	ND

a Data are shown as averages from three independent measurements. *K_D_*, equilibrium dissociation rate constant; *k_a_*, association rate constant; *k_d_*, dissociation rate constant; ND, not determined due to very fast kinetics and likely large errors in determining kinetics.

## DISCUSSION

A challenge for many viral pathogens, including HAdVs, is the penetration of the protective epithelial barrier (3, 21). The current model of HAdV entry into target cells involves fiber-mediated attachment to CAR on the cell surface and subsequent penton base-mediated internalization via RGD-binding integrins (46, 47). This model is largely based on usage of immortalized cell lines, which do not mimic polarized epithelia where CAR and integrins are not (or are to a limited extent) exposed on the apical side (48). Recent studies have indicated that the CAR_EX8_ isoform can be upregulated on the apical side of respiratory epithelial cells in the presence of neutrophils, which facilitate cellular entry of species C HAdVs (20, 21, 49). It has also been reported that the neutrophil chemoattractant IL-8 relocalizes the HAdV coreceptor α_V_β_3_ integrin to the apical surface of polarized epithelial cells. Interestingly, neutrophils are a major source for secretion of hLF in mucosa (50). Our results shed a novel light on these studies where neutrophils apparently play a role in HAdV entry. However, we demonstrate that neither fiber-CAR nor penton base-integrin interactions are engaged in hLF/Lfcin-mediated HAdV infection. Instead, we propose the presence of an alternative CAR- and integrin-independent mechanism as an important entry portal for HAdV-C5. In this model, hLF (or hLfcin) binds to both HAdV-C5 virions through charge-dependent interactions with hexon HVR-1 and serves as a bridge between the virus particle and the cell surface. Collectively, these interactions result in a 150- to 280-fold-increased transduction of CAR-negative and integrin-negative CHO-K1 cells and in 40-fold-increased infection of human respiratory epithelial cells that express high levels of CAR and integrins. This enhanced infection is likely to be even more prominent with hLfcin being constantly produced and present. *In vivo*, hLF and hLfcin are constantly present in the respiratory mucosa, and thus we can expect the effect of hLF/hLfcin-mediated infection to increase over time as the infection progresses.

We conclude that hLF/hLfcin1-49 mainly anchor to HVR-1 on the HAdV-C5 hexon, which binds to hLF with nM affinity and to hLfcin with μM affinity, which allows for enhanced transduction. However, we also noted that hLF and hLfcin are both able to prevent transduction if added at high, nonphysiological concentrations (15) (data not shown). This effect is likely due to a saturation of the system in which hLF/hLfcin not bound by the virus is able to engage the cellular receptor structure naturally used by hLF/hLfcin and block transduction. hLF differs from rhLF in glycosylation (hybrid type versus high-mannose type), but not in amino acid sequence (51, 52). hLF also differs from bLF (complex-type glycosylation) in both glycosylation and in amino acid sequence. Thus, the reason hLF (but not rhLF and bLF) promotes transduction is because (i) the glycans in rhLF (but not in hLF) shield and prevent LFcin from binding to hexon and/or to target cells or (ii) the hLF glycans (but not the rhLF glycans) stabilize LF binding to the hexon and/or to the target cell. Regardless of origin—human (hLfcin1-49) or synthesized (other Lfcins)—none of these peptides are glycosylated, and thus comparing the effect of these peptides is valid. HAdV-C5, HAdV-B35, and HAdV-A31 hexons all show very different affinities for hLF/hLfcin, which correspond to their different abilities to utilize hLfcin for infection. The hexon sequence is relatively well conserved between HAdVs and mainly differs in the HVRs. The most pronounced difference is observed in HVR-1 of species C, where HVR-1 is significantly longer and more negatively charged than those of other HAdVs (Fig. 6 and 7). Such an exposed and negatively charged loop may interact with the highly cationic domain of hLF/hLfcin. We verified this by the use of chimeric viral vectors in which HVR-1 of the HAdV-C5 hexon was replaced with the corresponding sequence of HAdV-A31. The infectivity of this chimeric vector was almost completely unaffected by hLfcin1-49. This strongly suggests that the major binding site for hLfcin1-49 on the virus capsid is HVR-1 of the hexon. However, as mutating HVR-7 (44) slightly affected transduction, we cannot rule out additional anchoring points for Lfcin on the hexon, but it may also be that this deletion disrupts Lfcin interaction with HVR-1. It should also be pointed out that we only used one concentration of the vectors, and it may be that the differences observed are concentration dependent. Furthermore, we show that hLfcin peptides enhance infection more efficiently than the full-length protein, despite a lower affinity of the hLfcin1-49-hexon interaction than that of the hLF-hexon interaction.

We propose that the trimeric arrangement of the hexons can accommodate up to three hLfcin1-49 molecules simultaneously and thereby increase the avidity. Additionally, the flat arrangement of the hexons within one face of the icosahedron may increase the avidity effect to the cell surface even further. Full-length hLF, on the other hand, is substantially larger, and there is only space for one single LF molecule on each hexon trimer. Unfortunately, we were not able to decipher the contribution of hLF with the hLfcin peptide cleaved off, as separation of the two species of LF is virtually impossible. However, in mucosa, the three species of hLF (hLfcin, hLF, and hLF without hLfcin) potentially coexist in a complex equilibrium that probably shifts during infection/inflammation (53). Even though additional mapping studies of hLfcin are required, our data also suggest that the length and secondary structure of hLfcin is of importance for the ability to enhance HAdV-C5 transduction. Even though the origins of the peptides differ (hLfcin1-49, cleaved from purified human tear fluid LF; other Lfcins, synthesized), we still think that the comparison of the peptides is relevant, since none of the Lfcin peptides (regardless of origin) are not posttranslationally modified.

Collectively our data suggest a “Trojan horse” mechanism by which selected HAdVs hijack the entry mechanism used by hLF. The viral capsid is first coated with hLF (or hLfcin) and subsequently engages a yet-to-be-determined receptor on the cell surface to enter target cells independent of an interaction with CAR. A crucial factor that determines whether HAdV can utilize this pathway or not is the amount of negative charge and the length of the first loop of the hexon protein (HVR-1). We suggest that this ability is a main mechanism whereby CAR-binding HAdVs, e.g., species C HAdVs, initiate infection at the apical side of polarized epithelial cells. For some CD46-binding HAdVs (e.g., HAdV-B35), this mechanism is likely to act in parallel to CD46, since CD46 is also expressed on the apical side (54). As the infection proceeds, progeny virions released from the basolateral side probably utilize CAR at a higher degree, as CAR is more abundant within the tissue (19). The ability to coat the capsid with hLF might also provide additional benefits beyond receptor engagement, such as escaping neutralizing antibodies, via a mechanism previously suggested for HAdV-C5 and coagulation factors (55).

Utilization of hLF/hLfcin by species C HAdVs could also account for the frequent and persistent infections of species C HAdVs in tonsil T lymphocytes in young children (56). We have previously shown that hLF facilitates HAdV-C5 infection of CAR-negative T cells (15). The levels of hLF in tonsils are not well studied, but bacteria that often colonize tonsils have been shown to be covered with hLF (57, 58), suggesting that hLF/hLfcin is prevalent in such tissues. Taken together, our results highlight the ingenuity of HAdVs and explain how they may ensure efficient infection in a very hostile and competitive environment, despite limited expression of CAR and integrins. In addition, such a Trojan horse mechanism could be of importance for additional viruses shown to infect mucosal surfaces with the aid of cell adhesion molecules.

## MATERIALS AND METHODS

### Cells, viruses, proteins, antibodies, and Lfcin peptides.

Human lung adenocarcinoma, A549, and CHO cells were grown according to ATCC guidelines. HAdV-C5 (strain Ad75), HAdV-B35 (strain Holden), and HAdV-A31 (strain 1315/63) were propagated in A549 cells with or without ^35^S-labeling according to Johansson et al. (59). HAdV-C5_GFP was obtained from Vector Development Laboratory, and mutated/chimeric HAdV-C5 vectors were described previously (43). Bovine (bLF) milk and recombinant hLF produced in Aspergillus awamori (rhLF) were obtained from Sigma and Baxter Pharmaceuticals Solutions, LLC, respectively. Human LF (hLF) was purified from human tear fluid (15) by size exclusion chromatography. DNA encoding fiber knobs from HAdV-C5 and HAdV-B35 was cloned to include the N-terminal trimerization motif (TLWT), expressed in Escherichia coli (Rosetta DE3) and purified either by Ni-nitrilotriacetic acid (NTA) bead (HAdV-C5) affinity chromatography (60). Anti-HAdV hexon antibodies were purchased from Merck Millipore (clone 20/11), RGS-His antibodies from Qiagen, and Alexa Fluor 488-conjugated secondary antibodies from Invitrogen. Bovine lactoferricin (bLfcin) 17-42 and human lactoferricin (hLfcin) 1-49 were purchased from the Center for Food Technology, processed by Hamilton, Qld, Australia. Briefly, human milk was collected from several donors and hLF purified. hLfcin was then generated by pepsin cleavage and purified as described previously (61). bLfcin17-31 and hLfcin18-42 were both synthesized by standard solid-phase peptide synthesis, while hLfcin18-42 was further cyclized in a two-step approach, as described previously (62). The truncated linear forms of Lfcin (1-49, 1-36, and 1-18) were purchased from GenScript.

### Virion binding experiments.

A549 cells were detached using phosphate-buffered saline (PBS) plus 0.05% EDTA with slow rocking at 37°C. Once detached, the cells were centrifuged at 1,500 × *g* for 5 min, resuspended in growth medium to about 2 × 10^6^ cells/ml, and allowed to recover at 37°C, slowly rocking to keep the cells in suspension. While recovering, the HAdV-Lfcin complex was set up. Lfcin (2 μM), or an equivalent volume of PBS, was mixed with ^35^S-labeled HAdV (15,000 particles/cell) in medium and left on ice at 4°C for 60 min. After 60 min of recovery, the cells were counted, and 150,000 viable cells were added to each well of a 96-V-shaped-well plate and centrifuged for 5 min at 1,500 × *g*. Once pelleted, the cells were resuspended in the HAdV-Lfcin solution or in HAdV-PBS solution and incubated on ice for 60 min at 4°C. After 60 min, the cells were pelleted and washed once in medium and once in PBS before the addition of scintillation fluid and quantification using a Wallac 1450 Microbeta counter (Trilux).

### Fiber knob binding experiments.

HAdV-C5 fiber knob-hLfcin complex was set up as follows: 15 μg/ml (2.5 μM) of hLfcin, or an equivalent volume of PBS, was mixed with 10 μg/ml of fiber knob in medium and left on ice at 4°C for 60 min. The recovered cells were counted, and 200,000 viable cells/well were plated as described above. Once pelleted, the cells were resuspended with fiber knob or fiber knob-hLfcin solutions, followed by binding on ice for 60 min at 4°C. Unbound fiber knobs were washed away with PF buffer (PBS supplemented with 2% fetal bovine serum [FBS]), and the cells were then incubated with an anti-RGS-His mouse monoclonal antibody on ice for 30 min. After washing, the cells were incubated with an Alexa 488-labeled anti-mouse antibody for 30 min on ice. Fiber knob-bound cells were analyzed with flow cytometry using a fluorescence-activated cell sorting (FACS) LSR II instrument (Becton, Dickinson). Results were analyzed using FACSDiva software (Becton, Dickinson).

### Transduction experiments.

For all transduction experiments, GFP-expressing vectors were used. The HAdV_GFP vector was purchased from Vector Development Laboratory. All mutated HAdV-C5 vectors were generated in the laboratory of A. Plückthun at the University of Zürich using the AdEasy system (Agilent) to contain an internal ribosome entry site (IRES)-luciferase (Luc)-GFP cassette for detection of transduction. To investigate the role of integrins in transduction, HAdV-C5-IRES-GFP with an intact RGD loop in the penton base protein (-DMNDHAIRGDTFATRAE-) and HAdV-C5-ΔRGD-IRES-GFP (-DMNDTSRAEEKRAE-), known not to bind integrins (40), were generated. To determine the binding site for hLF/hLfcin on the hexon protein, hexon-mutated vectors were produced with HVR-1 of HAdV-C5 (43) replaced with the corresponding sequence of HAdV-A31, as well as a HAdV-C5 vector mutated in HVR-7 (44).

For experiments in A549 cells, 15,000 cells were seeded into a black 96-well plate with a transparent bottom. For experiments with CHO-K1 cells, 20,000 cells were seeded. After 24 h, HAdV-C5 vectors was complexed with 2 μM Lfcin or with ∼12 μM (1 mg/ml) of LF and, after 60 min, added to the cells on ice. The blocking of CAR using soluble HAdV-C5 fiber knob was performed using 10 μg/ml of recombinant knob. After another 60 min, unbound complex was washed away using Dulbecco’s modified Eagle’s medium (DMEM), followed by final addition of fresh medium. After 44 h, the cells were fixed using 4% paraformaldehyde (PFA) for 10 min, and GFP expression was imaged using a Trophos system (Luminy Biotech Entreprises). For the hexon chimeric viruses, transduction was instead quantified by measuring the luciferase activity at 44 h postinfection using a Pierce luciferase glow assay kit.

### Long-term infection experiments.

A549 cells (30,000) were seeded into a 48-well plate in DMEM plus 5% FBS. The next day, the cells were infected by HAdV-C5, HAdV-B35, or HAdV-A31 in the presence or absence of 2 μM hLfcin1-49. The hLfcin1-49 complex was set up as described above, and binding to the cells occurred on ice for 60 min. Once unbound virus was removed by washing, fresh medium was added (DMEM plus 2% FBS). In wells infected with HAdV-hLfcin complex, hLfcin1-49 was added at a concentration of 1 μM. After 4 days, the supernatant was harvested, centrifuged, and added to a fresh monolayer of A549 cells. The collected supernatant was subjected to several 10-fold dilutions in DMEM before addition to the cells. Once added, the virus was allowed to bind to the cells for 60 min at 4°C. After binding, the cells were washed once, fresh DMEM plus 2% FBS was added, and the infection was allowed to run for 44 h at 37°C. Thereafter, the cells were fixed with 4% PFA for 10 min, followed by an additional fixation step using 100% methanol at −20°C. Staining of the HAdV hexon (MAB8052) was performed. After washing with PBS, an Alexa Fluor 488-conjugated secondary antibody was used to visualize the HAdV hexon. The stained plates were imaged using a Trophos system (Dioscure). To compare the amount of infectious virus produced, the lowest dilution was selected at which a complete monolayer was still intact.

### Purification of HAdV structural proteins.

Ten flasks of 70% confluent A549 cells were infected with HAdV-C5, HAdV-A31, or HAdV-B35. After initial inoculation, unbound virus was washed away, and approximately 30 ml of fresh medium (DMEM plus 20 mM HEPES [*N*-2-hydroxyethylpiperazine-*NN*-2-ethanesulfonic acid] plus 5% FBS) was added. At 48 h after infection, the cells were harvested by scraping into a small volume of DMEM, centrifuged, and resuspended in 6 ml of DMEM. The collected cells were lysed and the sample cleared by centrifugation. Virus was separated from soluble viral components by a CsCl gradient, as described previously (59). After ultracentrifugation, the top phase containing soluble viral components was recovered, and HAdV hexons were purified using monoclonal hexon antibodies (MAB8052) covalently attached to magnetic protein A/G beads (Pierce) using dimethyl pimelimidate (DMP) according to the manufacturer’s instructions. Bound hexons were washed three times using PBS to remove protein bound unspecifically, followed by elution in 50 μl of 200 mM glycine buffer at pH 2.5.

### Surface plasmon resonance.

CM5 sensor chips, an amine-coupling kit, and HBS-EP+ buffer (10 mM HEPES, 150 mM NaCl, 3 mM EDTA, and 0.005% [vol/vol] surfactant P20 [pH 7.4]) were all purchased from GE Healthcare. All surface plasmon resonance (SPR) experiments were performed at 25°C in HBS-EP+ running buffer with adenovirus hexons immobilized on the sensor surface. In addition, all experiments were performed with the same batch of LF/Lfcin. Data were collected with a Biacore T200 instrument at a rate of 1 Hz. HAdV hexons were coupled to the CM5 sensor chip by amine coupling reactions according to the manufacturer’s instructions, aiming for an immobilization density of 900 to 1,100 resonance units (RU). The surface of the upstream flow cell was used as a reference and was subjected to the same coupling reaction in the absence of protein. The analytes were serially diluted in running buffer to prepare a 2-fold concentration series ranging from 8 nM to 2 μM and then injected in series over reference and experimental biosensor surfaces for 120 sec at a flow rate of 30 μl/min. Blank samples containing only running buffer were also injected under the same conditions to allow for double referencing. After each cycle, the biosensor surface was regenerated with a 60-sec pulse of 10 mM Tris-glycine (pH 1.5) at a flow rate of 30 μl/min.

### Statistical analysis.

All experiments were performed at least three times in duplicates or triplicates. The results are expressed as means ± standard error of the mean (SEM), and two-way analysis of variance (ANOVA) or Student’s *t* test was performed using Prism version 7.00 for Windows (GraphPad Software, San Diego, CA). *P* values of <0.05 were considered statistically significant.
